# Association Study and Mendelian Randomization Analysis Reveal Effects of the Genetic Interaction Between *PtoMIR403b* and *PtoGT31B-1* on Wood Formation in *Populus tomentosa*

**DOI:** 10.3389/fpls.2021.704941

**Published:** 2021-08-30

**Authors:** Liang Xiao, Liting Man, Lina Yang, Jinmei Zhang, Baoyao Liu, Mingyang Quan, Wenjie Lu, Yuanyuan Fang, Dan Wang, Qingzhang Du, Deqiang Zhang

**Affiliations:** ^1^National Engineering Laboratory for Tree Breeding, College of Biological Sciences and Technology, Beijing Forestry University, Beijing, China; ^2^Key Laboratory of Genetics and Breeding in Forest Trees and Ornamental Plants, Ministry of Education, College of Biological Sciences and Technology, Beijing Forestry University, Beijing, China; ^3^Xining Forestry Science Research Institute, Xining, China

**Keywords:** PtomiR403b, *PtoGT31B-1*, association study, epistasis, Mendelian randomization, genetic interaction, *Populus*

## Abstract

MicroRNAs (miRNAs), important posttranscriptional regulators of gene expression, play a crucial role in plant growth and development. A single miRNA can regulate numerous target genes, making the determination of its function and interaction with targets challenging. We identified PtomiR403b target to *PtoGT31B-1*, which encodes a galactosyltransferase responsible for the biosynthesis of cell wall polysaccharides. We performed an association study and epistasis and Mendelian randomization (MR) analyses to explore how the genetic interaction between *PtoMIR403b* and its target *PtoGT31B-1* underlies wood formation. Single nucleotide polymorphism (SNP)-based association studies identified 25 significant associations (*P* < 0.01, *Q* < 0.05), and *PtoMIR403b* and *PtoGT31B-1* were associated with five traits, suggesting a role for PtomiR403b and *PtoGT31B-1* in wood formation. Epistasis analysis identified 93 significant pairwise epistatic associations with 10 wood formation traits, and 37.89% of the SNP-SNP pairs indicated interactions between *PtoMIR403b* and *PtoGT31B-1*. We performed an MR analysis to demonstrate the causality of the relationships between SNPs in *PtoMIR403b* and wood property traits and that *PtoMIR403b* modulates wood formation by regulating expression of *PtoGT31B-1*. Therefore, our findings will facilitate dissection of the functions and interactions with miRNA-targets.

## Introduction

Trees are an abundant renewable source of pulp and are important in the emerging bioenergy industry (Jansson and Douglas, 2007). Secondary cell walls form the bulk of woody tissue and affect wood quality and quantity (Zhang et al., 2014). Genetic analyses of annual herbals and perennial trees have showed that the cell wall comprises mainly cellulose, hemicellulose, and pectin, along with lignin and protein (Basu et al., 2016). The participation of genetic factors in the biosynthesis of cell wall components involves Galactosyltransferases and microRNAs (miRNAs) (Lu et al., 2013; Yu et al., 2014; Li et al., 2015; Fan et al., 2020).

Galactosyltransferases are encoded by a small gene family−glycosyltransferase 31 (GT31), include GALT and GALECTIN domains, and mediate the biosynthesis of cell wall polysaccharides, such as xyloglucan (XyG; the dominant component of hemicellulose) and rhamnogalacturonan I (RG-I; a pectin component) (Hennet, 2002; Jensen et al., 2012; Showalter and Basu, 2016; Matsumoto et al., 2019). The hemicellulose content of *Populus* wood is 16–23% on a dry weight (DW) basis, and the glucan content is 39–49% on a DW basis (Porth et al., 2013), suggesting an important role for galactosyltransferases in wood formation. But there are few functional analyses of galactosyltransferase genes in tree species, much of the research being conducted in *Arabidopsis thaliana* (Fagundes Lopes et al., 2010). For example, *GALT2* and *GALT5* function as AGP-Hyp-O-galactosyltransferases, and double mutants had altered phenotypes related to growth and development, including reduced silique length and plant height (Basu et al., 2015). *MURUS3* (*MUR3*) encodes a XyG-specific galactosyltransferase, which leads to dwarf mutants with short petioles and short inflorescence stems in *A. thaliana* (Kong et al., 2015). Overexpression of *EgMUR3* (the *MUR3* ortholog in *Eucalyptus grandis*) in *A*. *thaliana* resulted in similar phenotypes, implying a role for galactosyltransferase genes in wood formation (Fagundes Lopes et al., 2010).

MicroRNAs (miRNAs) are an endogenous class of *trans*-acting small non-coding RNAs (approximately 20–24 nucleotides) that are important posttranscriptional regulators of gene expression in eukaryotes (Ehrenreich and Purugganan, 2008). In plants, miRNAs play roles in numerous biological processes (Budak and Akpinar, 2015). For instance, miRNAs are implicated in wood formation in *Populus*—PtomiR397a downregulated the expression of *LACs* and reduced the Klason lignin content by as much as 22% in *Populus trichocarpa* (Lu et al., 2013). In addition, PtomiR6443 regulated *Ferulate 5-hydroxylase* (*F5H*) to alter lignin composition and enhance saccharification in *Populus tomentosa* (Fan et al., 2020). However, no systematic effort has been made to characterize the miRNAs that regulate the galactosyltransferase genes.

Association studies enable the identification of DNA variants associated with phenotypic variation, especially for the quantitative traits of perennial trees because of their abundant genetic variants and the large number of genomes sequenced (Cardon and Bell, 2001; Neale and Savolainen, 2004). This strategy has been used to identify single nucleotide polymorphisms (SNPs) associated with wood characteristics in several perennial tree species. For instance, Wegrzyn et al. (2010) explored the effects of SNPs in lignin and cellulose biosynthesis genes on wood chemistry traits and identified the polymorphisms responsible for phenotypic variation in *P. trichocarpa*. In Mendelian randomization (MR) analyses, a causal relationship between two heritable complex traits is inferred, with one reflecting exposure and the other taken as the outcome (Burgess et al., 2018). MR tests is a widely method to assess the causal relationship between complex traits and environmental factors or gene expression (Porcu et al., 2019). For example, MR studies have identified causal relationships between specific gene expression and clinical traits, with gene expression treated as an exposure risk factor for the manifestation of complex traits, indicating that MR analyses can bridge the causal relationship between genetic variation, gene expression, and complex traits (Li et al., 2016; van der Graaf et al., 2020). MiRNAs are *trans*-regulators of gene expression, implying that MR can be used to uncover causal relationships between miRNAs and desirable traits. Therefore, by combining an association study and a MR analysis, insight into how genetic interactions between miRNAs and their targets affect desirable traits can be obtained.

We report here that PtomiR403b, a conserved miRNA, was highly expressed in the developing xylem. An association study and epistasis analysis were conducted to explore the genetic effects of PtomiR403b and its target *PtoGT31B-1* on tree growth and wood formation in an association population of *P. tomentosa*, and an MR analysis was performed to assess the causative relationship between *PtoMIR403b* and *PtoGT31B-1* underlying the wood characteristic traits. Collectively, our aim was to identify significant SNPs in *PtoMIR403b* that are associated with wood characteristic traits, analyze the genetic interaction between *PtoMIR403b* and *PtoGT31B-1*. Ultimately, our findings provide a strategy to characterize the genetic interaction of miRNA and its targets, and also contribute to the improvement of *Populus* wood yield and quality via marker-assisted breeding.

## Materials and Methods

### Association Population and Phenotypic Data

#### Association Population

The *P*. *tomentosa* association population used in this study consisted of 435 unrelated individuals representing almost the entire natural distribution (30–40°N, 105–125°E). The accessions were cloned via root segments in a randomized complete block design with three blocks in 2009 in Guan Xian County, Shandong Province, China (36°23′N, 115°47′E). The total genomic DNA from each accession was extracted from fresh leaves of each individual using a DNeasy Plant Mini kit (Qiagen, Shanghai, China) following the manufacturer’s protocol.

#### Phenotypic Data

We measured 10 wood characteristic traits for the 435 individuals of *P. tomentosa*—>diameter at breast height (DBH), tree height (H), stem volume (V), α-cellulose content (AC), holocellulose content (HC), hemicellulose content (HEC), lignin content (LC), fiber length (FL), fiber width (FW), and microfiber angle (MFA). Measurements were conducted following the method described in Du et al. (2014).

### Identification and Isolation of *PtoMIR403b* and Its Target Genes

To clone the full-length sequence of *PtoMIR403b* in *P. tomentosa*, we used gene-specific primers based on the primary sequence of PtomiR403b, which contains the pre-miRNA region of the *PtoMIR403b* sequence and 600 bp of flanking region on each side. Next, psRNATarget^1^ was used to predict putative target genes of PtomiR403b in the genome-wide transcript of *P. tomentosa*, with the expectation cutoff set to 2.0. In addition, degradome sequencing of pooled samples of six tissues (leaf, shoot apex, phloem, cambium, developing xylem, and mature xylem) was performed to identify potential cleavage sites and verify the psRNATarget results, in which *PtoGT31B-1* was identified as a putative target of PtomiR403b. Finally, we cloned the full-length sequence of *PtoGT31B-1* from the genome of *P. tomentosa*.

Degradome sequencing enables the identification of miRNA cleavage sites in target genes via the sequencing of RNA ends. We performed degradome sequencing using equal pooled RNA samples of six tissues (leaf, shoot apex, phloem, cambium, developing xylem, and mature xylem) from *P. tomentosa*. The pooled RNA samples were used with biotinylated random primers to build a degradome-sequencing library as described previously (German et al., 2008). MiRNA cleavage sites were identified using the CleaveLand pipeline based on *P. tomentosa* genome transcripts (Addo-Quaye et al., 2009). The detailed methods have described in Supplementary Methods 1.

### RNA Ligase-Mediated 5′ Rapid Amplification of cDNA Ends

To identify cleavage sites in target genes, RNA ligase-mediated 5′ rapid amplification of cDNA ends (RLM-5′ RACE) was conducted using the SMARTer RACE Kit (TaKaRa, Shiga, Japan) in accordance with the manufacturer’s instructions with modifications. Briefly, extracted total RNA was ligated to a 5′ RACE adapter using T4 RNA ligase, followed by cDNA template synthesis via reverse transcription with 5′-RACE CDS Primer A [5′-(T)25 V N-3′; N = A, C, G, or T; V = A, G, or C]. Next, 5′ RACE PCR was performed using a universal primer (forward) and a gene-specific primer (reverse, Supplementary Table 1), with cDNA as the template. The products were gel purified, cloned, and sequenced.

### Real-Time Quantitative PCR

To evaluate the expression of PtomiR403b and its targets, we used the phloem, cambium, developing xylem, mature xylem, leaf, and shoot apex tissues of a 1-year-old *P. tomentosa* clone. Total RNA was extracted using the Plant Qiagen RNeasy Kit (Qiagen China, Shanghai) following the manufacturer’s instructions and purified using the RNase-Free DNase Set (Qiagen). mRNAs were reverse transcribed into cDNA using the Reverse Transcription System (Promega Corporation, Madison, WI) according to the manufacturer’s instructions. MiRNAs were reverse transcribed into cDNA using the miRcute Plus miRNA First-Strand cDNA Synthesis Kit (Tiangen, Beijing, China). Gene-specific primers were used for real-time quantitative PCR (RT-qPCR) on the 7500 Fast Real-Time PCR System with SYBR Premix Ex Taq (TaKaRa) and the miRcute Plus miRNA qPCR Kit (SYBR Green; Tiangen) (Supplementary Table 1). All reactions were performed with three technical and biological replicates with poplar *actin* (accession number: EF145577) used as the internal control. The PCR amplification program (Xiao et al., 2017) was as follows: initial denaturation at 94°C for 5 min; 40 cycles of 94°C for 30 s, 58°C for 30 s, and 72°C for 30 s; and a final melting curve from 70 to 95°C.

### Phylogenetic Analysis of miR403

For the phylogenetic analysis of miR403, we downloaded all precursor sequences of miR403 from miRbase,^2^ thus obtaining 40 members from 22 species (including four members of PtomiR403). We used Muscle in MEGA ver. 7.0 software with the default settings to perform multiple sequence alignments (Kumar et al., 2016). Phylogenetic trees were constructed using the maximum-likelihood method in MEGA ver. 7.0 software; branch support was estimated with 1,000 bootstrap replicates. Figtree software^3^ was used to visualize the phylogenetic tree.

### Identification of SNPs in *PtoMIR403b* and *PtoGT31B-1*

The association population of 435 accessions was resequenced on the Illumina GA2 sequencing platform at an average depth of 15 × genome coverage (raw data). To obtain the clean data, the Raw reads were trimmed through a series of quality control (QC) procedures. QC standards as the following: (1) Removing reads with ≥ 10% unidentified nucleotides (N); (2) Removing reads with > 50% bases having phred quality < 5; (3) Removing reads with > 10 nt aligned to the adapter, allowing ≤ 10% mismatches; (4) Removing putative PCR duplicates generated by PCR amplification in the library construction process (read 1 and read 2 of two paired-end reads that were completely identical). Then, the clean reads were mapped to the *P. tomentosa* reference genome and used for SNP calling. VCFtools software was used to extract gene-derived biallelic SNPs from the full-length sequences of *PtoMIR403b* (including the pre-miRNA and 600-bp flanking sequences on each side) and *PtoGT31B-1* (including the 2-kb upstream promoter sequence and 500-bp downstream flanking sequence). We identified 54 and 165 high-quality SNPs in *PtoMIR403b* and *PtoGT31B-1*, respectively, with a minor allele frequency of > 5% and a miss rate of < 20% across 435 accessions. The detailed methods described in the Supplementary Method 2.

### Nucleotide Diversity Analysis and Linkage Disequilibrium Test

To evaluate nucleotide diversity, we estimated π (the average number of pair-wise differences per site between sequences) and θw (the average number of segregating sites per site) using Tassel ver. 2.0 software. For linkage disequilibrium (LD) analysis, we calculated the squared correlation of allele frequencies (*r*^2^) between pairs of SNPs in *PtoMIR403b* and *PtoGT31B-1*. To assess the pattern of LD in *PtoMIR403b* and its target, the decay of LD with physical distance (base pairs) within each SNP was estimated in 10^5^ permutations of the genotype data using non-linear regression. Singletons were excluded from the LD analysis.

### Single SNP-Based Association Analysis

A single SNP-based association analysis was performed for all SNP-trait associations between 219 common SNPs and 10 traits using Tassel ver. 5.0 software with a mixed linear model (MLM) that controls for kinship coefficients (*K*) and population structure (*Q*) (Bradbury et al., 2007). The *K* and *Q* matrices were obtained as described by Du et al. (2019). The QVALUE package in R was used to correct for multiple testing based on the positive false discovery rate method. The significance threshold for a single SNP-based association was defined as *P* < 0.01 and *Q* < 0.05.

### Multi-SNP Epistasis Association Analysis

Multifactor Dimensionality Reduction (MDR) ver. 3.0.2 software was used to detect epistatic effects among the SNPs (Hahn et al., 2003). The RelifF algorithm in MDR 3.0.2 was used to improve the reliability of probability approximation by filtering all unlinked SNPs (*r*^2^ < 0.1 or different genes) and identify the best five loci for each trait. An entropy-based measure was used to detect significant interactions between SNP-SNP pairs and calculate information gains (IGs) to evaluate epistatic effects.

### Expression Level of *PtoGT31B-1* in a Natural Population of *P. tomentosa*

Developing xylem tissues from the 435 accessions in a natural population of *P. tomentosa* were collected. Total RNA was extracted, reverse transcribed into cDNAs, and subjected to RT-qPCR to assess the expression level of *PtoGT31B-1* in 435 accessions of *P. tomentosa*. All reactions were performed with three technical replicates with poplar *actin* as the internal control. Relative mRNA levels were calculated using the comparative threshold cycle method.

### Mendelian Randomization (MR) Analysis

MR analysis was performed to evaluate the causality of the relationships between genetic variants and traits. To estimate the genetic effects of SNPs on the expression of *PtoGT31B-1*, we conducted an association analysis of the SNPs and *PtoGT31B-1* expression in the 435 accessions using Tassel 5.0 software with an MLM. The SNPs significantly associated with wood characteristic traits were subjected to MR analysis. The MendelianRandomization package in R was used for MR analysis with the inverse-variance weighting (IVW) method to summarize the effects of multiple SNPs (Yavorska and Burgess, 2017).

## Results

### Identification of PtomiR403b and Its Potential Targets in *P. tomentosa*

The miRNA transcription profiles in the leaf, mature xylem, cambium, developing xylem, shoot apex, and phloem tissues of *P. tomentosa*, indicated that miR403b was highly expressed in the shoot apex and phloem, suggesting a putative role for miR403b in *Populus* growth and development. Thus, we selected PtomiR403b for further analysis. We isolated the 1,300-bp primary sequence of *PtoMIR403b* from *P. tomentosa* containing a 21-bp mature region, 100-bp pre-miRNA region, and 600-bp flanking sequences around the pre-miRNA region. Prediction of the secondary structure of the precursor of PtomiR403b using RNAfold^4^ revealed a typical stem-loop structure, verifying that PtomiR403b is a miRNA (Supplementary Figure 1).

**FIGURE 1:**
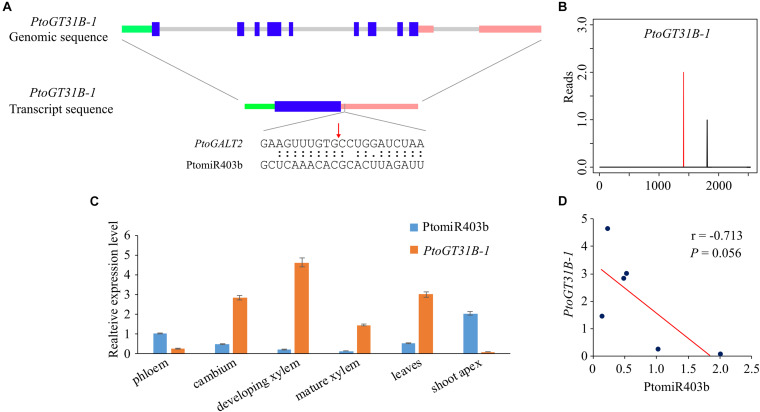
Identification of PtomiR403b and its target gene *PtoGT31B-1*. **(A)** Structure of *PtoGT31B-1* (top) and the transcript of *PtoGT31B-1* (middle); bottom, binding site of PtomiR403b. Green, blue, pink, and gray lines indicate the 5′-UTR, exon, 3′-UTR, and intron regions, respectively. The red arrow indicates the cleavage site confirmed by RLM-5′ RACE. **(B)** PtomiR403b cleavage sites in *PtoGT31B-1* as revealed by degradome sequencing. Red vertical bars indicate the most likely cleavage sites. **(C)** Expression pattern of PtomiR403b and its target *PtoGT31B-1* in six tissues. **(D)** Correlation of PtomiR403b and *PtoGT31B-1* expression. r, Pearson correlation coefficient (two-tailed test).

To identify the target genes of PtomiR403b, we used the psRNATarget to predict the putative targets in the genome-wide transcripts of *P. tomentosa* identified Ptom.002G.02518 (encoding β-1,3-galactosyltransferase 2 isoform X1, *PtoGT31B-1*), which is cleaved by PtomiR403 with an expectation value of ≤ 2. *PtoGT31B-1* is a member of CAZy GT-family-31 and mediates the synthesis of β-(1,3)-Gal. Degradome sequencing verified *PtoGT31B-1* as a target of PtomiR403b. 5′-RACE confirmed that PtomiR403b cut *PtoGT31B-1* at 1,449 nt in the 3′-untranslated region (UTR) (Figure 1A). The *PtoGT31B-1* cDNA is 2,540 bp long, with a coding region of 969 bp (322 amino acids) flanked by a 440-bp 5′-UTR and an 1131-bp 3′-UTR (Figure 1B).

miR403 has diverse functions in different species. Therefore, we aligned all precursor sequences of miR403 from miRbase, which included 40 members from 22 species. Mature sequences were conserved across 18 species, whereas the number of miR403 members varied among species, with some absent from eudicots, suggesting that the functional role of miR403 has changed over the course of plant evolution (Supplementary Figure 2). Phylogenetic analysis showed that PtomiR403 exhibits high homology with miR403 from *P. trichocarpa*, indicating that the miR403 family is conserved in *Populus*.

### Tissue Specific Expression Pattern Reveal the Negative Correlation Between PtomiR403b and *PtoGT31B-1*

RT-qPCR showed that PtomiR403b and *PtoGT31B-1* expression varied among the eight different tissues of *P. tomentosa*. PtomiR403b expression was highest in the shoot apex, followed by the phloem, and lowest in mature xylem (Figure 1C). By contrast, *PtoGT31B-1* expression was highest in developing xylem, followed by leaves, and lowest in the shoot apex, implicating that *PtoGT31B-1* involving in wood formation. The correlation between the expression of PtomiR403b and its target was significantly negative (Pearson *r* = −0.713, *P* = 0.056), suggesting that PtomiR403b might negatively regulated the expression of *PtoGT31B-1* (Figure 1D).

### *PtoMIR403* and *PtoGT31B-1* Exhibited High Nucleotide Diversity and Rapidly Declining LD

Genomic resequencing of 435 *P. tomentosa* accessions identified 54 and 165 common SNPs in *PtoMIR403b* and *PtoGT31B-1*, respectively (Supplementary Table 2). For *PtoMIR403b*, no SNP was identified in the mature miRNA region, whereas three were detected in the precursor regions. We predicted the effect on the stem-loop structure and minimum free energy (MFE) of the SNPs in the precursor regions. PtoMIR403b_SNP31 and PtoMIR403b_SNP33 significantly altered the stability of secondary structure, whereas PtoMIR403b_SNP32 did not affect the stem-loop structure and MFE (Supplementary Figure 1). Moreover, these two deleterious variants had high LD (*r*^2^ = 0.948) and affected the stem-loop structure more significantly. In addition, the primary sequence nucleotide diversity was higher than that of the precursor sequence, indicating different selective pressures in these regions. For *PtoGT31B-1*, the average synonymous diversity (*d*_*S*_) of the coding region was higher than the non-synonymous diversity (*d*_*N*_), with a *d*_*N*_/*d*_*S*_ ratio of < 1 (0.83), indicating that the non-synonymous sites had experienced purifying selection. In addition, we also found that the nucleotide diversity of *PtoGT31B-1* (π = 0.057) was higher than that of *PtoMIR403b* (π = 0.014), implying that *PtoMIR403b* and *PtoGT31B-1* experienced different selection pressures.

The squared allelic correlation coefficient (*r*^2^) between common SNP pairs was calculated to evaluate the overall patterns of LD for *Pto-MIR403b* and *PtoGT31B-1*. Non-linear regression showed that the LD decayed rapidly, decreasing to 0.1 within about 500 bp for *Pto-MIR403b* and about 2,500 bp for *PtoGT31B-1*. Therefore, the LD of *Pto-MIR403b* and *PtoGT31B-1* does not extend to the over entire gene sequences (Supplementary Figure 3).

### Allelic Variation of *PtoMIR403b* and *PtoGT31B-1* Allelic Variation Affects Tree Growth and Wood Formation

To explore the effects of *PtoMIR403b* and its target *PtoGT31B-1* on tree growth and wood formation, we measured 10 wood characteristic traits of 435 individuals in a natural population of *P. tomentosa* that exhibited high phenotypic diversity. An association analysis to test the additive/dominant effects between SNPs in *PtoMIR403b* and its target gene and 10 traits using a MLM in Tassel 5.0 software. At the threshold of *P* < 0.01 and *q* < 0.05, we identified 25 significant associations, corresponding to 15 SNPs and 8 traits (Supplementary Table 3). Each SNP explained 0.61–16.35% of the phenotypic variance (*R*^2^), with an average *R*^2^ of 7.30%. Among the 25 significant associations, 10 out of 25 associations showed additive effects, 19 exhibited dominant effects, and four presented both additive and dominant effects.

**FIGURE 2:**
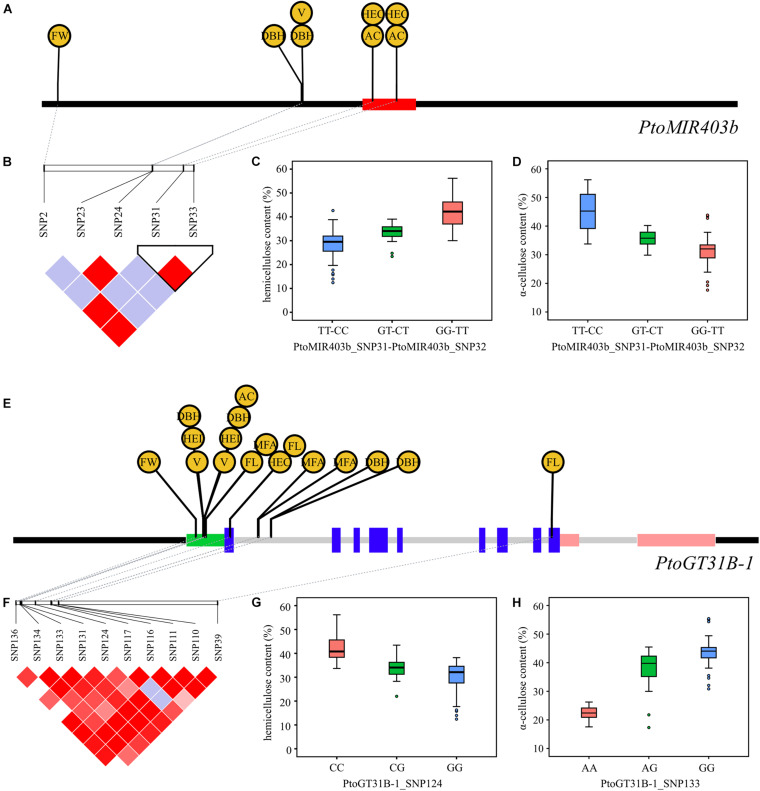
Allelic loci in *PtoMIR403b* and *PtoGT31B-1* significantly affecting wood formation. **(A)** SNPs in *PtoMIR403b* significantly associated with wood characteristic traits. **(B)** Haploview plot of the LD coefficient *r*^2^ for pairs of significantly associated SNPs in *PtoMIR403b*. PtoMIR403b_SNP31 and PtoMIR403b_SNP32 had a high LD coefficient *r*^2^. **(C,D)** Combined genotypic effect of PtoMIR403b_SNP31 and PtoMIR403b_SNP32 on hemicellulose content **(C)** and α-cellulose content **(D)**. **(E)** SNPs in *PtoGT31B-1* significantly associated with wood characteristic traits. **(F)** Haploview plot of the LD coefficient *r*^2^ for pairs of significantly associated SNPs in *PtoGT31B-1*. **(G)** Genotypic effect of PtoGT31B-1_SNP124 on hemicellulose content. **(H)** Genotypic effect of PtoGT31B-1_SNP133 on α-cellulose content.

For *PtoMIR403b*, we detected the deleterious variants PtoMIR403b_SNP31 and PtoMIR403b_SNP33 in the precursor region of *PtoMIR403b*; each had a high LD that altered the stem-loop structure of PtomiR403b. As expected, these two SNPs were significantly associated with HEC and AC, indicating that allelic variation in *PtoMIR403b* affects wood formation (Figures 2A–D). *PtoMIR403b* and *PtoGT31B-1* were both associated with five traits, supporting a shared role of *PtoMIR403b* and *PtoGT31B-1* involving in the same regulatory pathway. For instance, PtoGT31B-1_SNP124 located in the exon region of *PtoGT31B-1* significantly associated with the HEC and PtoGT31B-1_SNP133 situated in the 5′-UTR that associated with the AC, and PtoMIR403b_SNP31 and PtoMIR403b_SNP33 significantly associated with both HEC and AC, indicating that PtomiR403b and its target gene co-regulate wood formation (Figures 2E–H). We also detected SNPs in *PtoGT31B-1* associated with the other seven traits of wood formation, implying the important role of *PtoGT31B-1* in wood formation.

### Pairwise Epistasis Revealed the Allelic Interaction Between *PtoMIR403b* and *PtoGT31B-1*

**FIGURE 3:**
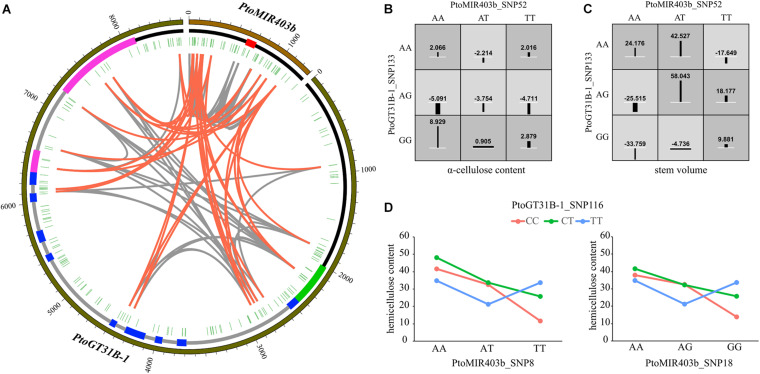
Epistatic interaction between *PtoMIR403b* and *PtoGT31B-1* underlying wood formation. **(A)** Circos plot representing SNP pairwise interactions affect wood characteristic traits. The outer, middle, and inner circles show the length, structure, and location of each SNP, respectively, in *PtoMIR403b* and *PtoGT31B-1*. Interior lines represent the pairwise interactions underlying 10 wood characteristic traits. Gray and orange lines indicate intra-gene interactions and inter-gene interactions between SNP pairs, respectively. **(B,C)** Epistatic effects of the interaction between PtoMIR403b_SNP52 and PtoGT31B-1_SNP133 on a-cellulose content **(B)** and stem volume **(C)**. **(D)** Epistatic effects on hemicellulose content of PtoGT31B-1_SNP116 and two loci from *PtoMIR403b*.

To assess the epistasis interactions between *PtoMIR403b* and its target gene *PtoGT31B-1*, we conducted epistatic analysis between each SNP pairs for 10 growth and wood formation traits via MDR software. Collectively, 95 significant pairwise epistatic associations were identified for 10 wood formation traits, including 14 unique SNPs in *PtoMIR403b* and 18 unique SNPs from *PtoGT31B-1* (Supplementary Table 4). The pairwise epistatic effects ranged from 0 to 10.34% (Figure 3A). Eight SNP-SNP pairs were associated with more than one trait. Moreover, the information gains (IGs) were used to estimate the mode of action of the epistatic interactions of the SNP-SNP pairs. We found that the IGs ranged from − 0.767 to 0.0437%, and most (86.32%) had negative IGs, indicating that the SNPs involved in the same process with functional overlap, and showed redundancy of genetic effects. For example, the PtoGT31B-1_SNP133-PtoGT31B-1_SNP114 exhibited epistasis effects on HC and V with different IGs of − 0.0273 for HC and 0.0437 for V (Figures 3B,C). The contrasting IGs for this SNP-SNP pair indicate the differential effects of epistatic interactions on growth and wood formation. Among all epistatic interactions, 37.89% showed the interaction between the *PtoMIR403b* and *PtoGT31B-1*; the remainder were intragenic SNP-SNP interactions. PtoGT31B-1_SNP19 interacted with four SNPs in PtomiR403b that showed epistatic effects on FW, implying the genetic interactions between PtomiR403b and *PtoGT31B-1*. The SNP-SNP pairs also exhibited pleiotropy, with eight SNP-SNP pairs contributing to at least one trait. For example, PtoMIR403b_SNP52 interacted with PtoGT31B-1_SNP133, which is responsible for phenotypic variation in HC and V. Only three SNPs of epistasis were detectable with additive or dominant effects, indicating that these SNP–SNP pairs exhibited more substantial epistatic effects than single SNPs.

To investigate the effects of single SNPs and SNP-SNP pairs underlying tree growth and wood properties, we focused and constructed the interaction graphs for HEC. We detected six SNP-SNP pairs showed epistatic effects on HEC, including four unique SNPs. PtoGT31B-1_SNP116 had a dominant effect on MFA and epistatic interactions with three SNPs associated with HEC, including two SNPs from PtoMIR403b. For example, we observed the different phenotypic values between genotypic combinations of PtoGT31B-1_SNP116 and PtoMIR403b_SNP18 led to different outcomes, with the AA-CC genotypic combination showed the highest phenotypic values of HEC (Figure 3D), indicating the epistasis effects of SNP pairs significantly contribute to the variation in HEC.

### Mendelian Randomization Test Revealed That the *PtoMIR403b* Associated With Wood Formation by Regulating the Expression of *PtoGT31B-1*

**FIGURE 4:**
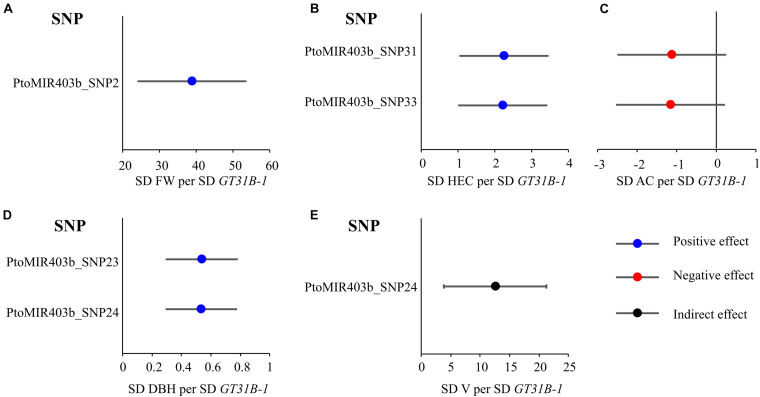
Estimates of the genetic effects of SNPs in *PtoMIR403b* on the expression of *PtoGT31B-1* and wood characteristic traits. Estimates were derived via MR using the IVW method. Estimates of effects on FW **(A)**, HEC **(B)**, AC **(C)**, DBH **(D)**, and V **(E)** relative to *PtoGT31B-1* (*GT31B-1*) expression.

Based on the association results of *PtoMIR403b* and the gene expression of *PtoGT31B-1* of these accessions, we performed MR test to identify traits whose variation is relevant to *PtoMIR403b* via affect the expression of target. First, we investigated the effects of causal SNPs within the *PtoMIR403b* for expression of *PtoGT31B-1*. Next, the genetic effects of significant SNPs in *PtoMIR403b* for expression of *PtoGT31B-1* and wood characteristics trait were integrated to perform MR test. The results of single-SNP based association have identified five SNPs which significantly associated with five traits, so we performed MR analysis using the IVW method of MR analysis to estimate the genetic effects arising from the expression of *PtoGT31B-1* on its corresponding trait. We identified that four of five traits were affected by the expression of *PtoGT31B-1*, which included expression of *PtoGT31B-1* positively contribute to FW, HEC, AC, and DBH trait, and expression of *PtoGT31B-1* negatively contribute to AC trait (Supplementary Table 5 and Figure 4). Also, 80% of the associations have proved the causal relationships between SNPs and traits, indicating that the genetic variation in *PtoMIR403b* contributes to wood formation by modulating expression of *PtoGT31B-1*. Interestingly, we found that SNPs in the precursor region (which altered the stem-loop structure) for expression of *PtoGT31B-1* had a positive effect on HEC content, and had a negative effect on AC content. These results support our analytic strategy and the statistical power of the MR analysis to dissect the function of miRNAs and indicate that PtomiR403b post-transcriptionally regulates *PtoGT31B-1* to modulate wood formation.

### Transcript Analysis of a Significant Haplotype Allele of *PtoMIR403b*

The results of association studies and MR test showed that a haplotype (PtoMIR403b_SNP31 and PtoMIR403b_SNP33) of *PtoMIR403b* significantly altered the expression of *PtoGT31B-1* and affected the HEC content. To further explore the effects of haplotype in the precursor region on the expression level of PtomiR403b and *PtoGT31B-1*, we next assayed the relative expression levels of PtomiR403b and *PtoGT31B-1* in developing xylem from 10 randomly selected individuals for each allele of the causal SNPs in the association population of *P. tomentosa*. The transcript abundances of PtomiR403b and *PtoGT31B-1* differed significantly among the alleles and exhibited opposite expression patterns (Supplementary Figure 4). PtomiR403b expression was high for the GG-TT allele but low for the TT-CC allele. By contrast, *PtoGT31B-1* expression was higher for the TT-CC allele than for the GG-TT allele, indicating that PtomiR403b downregulates *PtoGT31B-1* expression.

## Discussion

Wood formation is a complex trait of perennial tree species. Cellulose, hemicellulose, and lignin, the major components of wood tissue, have favorable relative proportions and structures in *Populus* wood (Porth et al., 2013). Genetic modification of genes involved in the biosynthesis of woody components can alter the woody growth and biomass of plants. In this study, we identified a conserved miRNA, PtomiR403b, which modulates wood formation by regulating its target *PtoGT31B-1*, a member of the galactosyltransferase family. Integration of association studies and Mendelian randomization test, we investigated the allelic interactions between *PtoMIR403b* and *PtoGT31B-1* underlying wood formation. Notably, we identified two SNPs in the precursor region of PtomiR403b that contributed to HC variation by regulating the expression of *PtoGT31B-1*. We systematically explored the genetic interaction between PtomiR403b and its target *PtoGT31B-1* and identified the causal alleles responsible for wood formation, providing a theoretical basis for molecular-assisted breeding of *Populus*.

### Characterization of PtomiR403b and Its Target PtoGT31B-1 in *Populus*

Wood formation in tree is highly plastic and requires the integration of complex developmental pathways. Molecular genetics studies in *Populus* have shown that woody growth is driven primarily by secondary cell wall formation. The secondary cell wall is predominantly composed of cellulose, hemicelluloses, and lignin. The biosynthesis genes for cellulose, hemicelluloses, and lignin have been characterized, as have those responsible for the supply of sugars and other secondary wall biosynthetic pathway precursors such as cellulose synthases (*CESAs*), cinnamoyl coenzyme A reductase (*CCR*), and trichome birefringence-likes (*TBLs*) (Zhong and Ye, 2014). In the recent years, the exhaustive data of RNA-seq from woody tissues have improved our understanding of transcriptional regulation during wood formation. Especially, miRNA as a short endogenous non-coding RNAs that posttranscriptionally regulate gene expression and are involved in various physiological processes. *In silico* analyses have identified numerous miRNAs involved in wood formation. Genetics studies in *Populus* have also characterized several miRNAs modulate the biosynthesis of woody components through its target genes. For example, Ptr-miR397a specifically down-regulated the expression of *laccases* (*LACs*), thus reducing lignin content (Lu et al., 2013), and PtomiR6443 alters lignin composition during stem development by modulating the expression of its target gene, *F5H*, which encodes the limiting enzyme in the biosynthesis pathway of sinapyl (S) monolignol (Fan et al., 2020). We found that miR403b regulates *PtoGT31B-1* in *Populus*, possibly modulating wood formation.

We found that miR403 is a conserved miRNA family in eudicots whose member number varies among species and is absent from 22 species in miRBase (Supplementary Figure 2). We identified four miR403 copies in *Populus* but only one in *Arabidopsis* with the same mature sequence. Surprisingly, miR403 targets AGO2 in *Arabidopsis* and is restricted to few plant species, whereas the target gene encoding AGO2 was lost in *Populus*, suggesting that the function of miR403 has been adapted during the course of plant evolution. Previous studies have predicted that miR403b target six genes in *Populus* (Xie et al., 2017), we focused on the interaction of miR403b-GT31B-1 to assess the role of miR403b in wood formation. The results of degradome sequencing, 5′-RACE, and expression patterns provided the evidence that miR403b cleave the *PtoGT31B-1*, suggesting *PtoGT31B-1* was the target of PtomiR403b (Figure 1). *PtoGT31B-1* is a member of the galactosyltransferase family glycosyltransferase 31 and harbors GALT and GALECTIN domains. This family is responsible for the biosynthesis of cell wall components, such as hemicelluloses and pectin. For instance, *AtGALT9* modulates cell wall pectin content, thereby controlling organ size (Zhang et al., 2021). An association study and MR testing indicated a causal relationship between PtomiR403b-*GT31B-1* and wood characteristic traits. Therefore, PtomiR403b targets *PtoGT31B-1* to modulate wood formation in *Populus*.

Moreover, we also found that the mature region of *PtoMIR403b* lacked SNPs and was conserved in the natural population of *P. tomentosa*. A haplotype block (including two SNPs) in the precursor region significantly altered the stem-loop structure and increased the MFE of pre-miR403b (Supplementary Figure 1). The transcript abundance of PtomiR403b differed significantly across the three genotypes (Supplementary Figure 4). The two SNPs in the precursor region were deleterious variants that alter the secondary structure and depress the generation of mature PtomiR403b, indicating that SNPs in the pre-miRNA region can have regulatory functions. SNPs in the precursor region can affect miRNA biogenesis, and most studies support the notion that causal SNPs in miRNAs can contribute to phenotypic variation (Xie et al., 2017). In this study, PtoMIR403b_SNP31 and PtoMIR403b_SNP33 had the top two association signals for HC and altered the expression of *PtoGT31B-1* to modulate HC variation. In addition, SNPs in *PtoMIR403b* were associated with DBH, V, and FW, implying a role for PtomiR403b in wood formation.

### Allelic Variations in *PtoMIR403b* and *PtoGT31B-1* Affect Wood Formation in *Populus*

Ample study has revealed that SNPs in miRNA genes can alter miRNA biogenesis and function miRNA (Cai et al., 2009). Due to the SNPs may recruit or affect the combination of the miRNA to its target genes, thereby influence the regulation effects, and associate with the phenotypic variation (Mishra et al., 2008; Cai et al., 2009). For instance, Ehrenreich and Purugganan (2008) characterized the SNPs and nucleotide divergence in miRNAs and their binding sites in *Arabidopsis*. Several SNPs were predicted to affect the secondary structure of pre-miRNA. An SNP in the mature region of *PtoMIR6466* that alters the stem-loop structure and was associated with photosynthetic traits (Xiao et al., 2020). Herein, we identified five SNPs in PtoMIR403b significantly associated with wood characteristic traits, suggesting a role for PtomiR403b in wood formation (Figure 2). A haplotype (including two SNPs) in the precursor region was significantly associated with HC. Secondary structure prediction showed that the haplotype alters the stem-loop structure and increases the MFE of pre-miR403b. We also detected a different transcript abundance of PtomiR403b and its target *PtoGT31B-1* differed among the three genotypes. Therefore, this haplotype alters the secondary structure and depresses the generation of mature PtomiR403b, thus contributing to HC variation in *Populus*. MR test also revealed that this haplotype regulates the expression of its target *PtoGT31B-1* to modulate variation in HC. The PtoMIR403b_SNP31 and PtoMIR403b_SNP33 haplotype was a favorite allele combination for the marker-assisted breeding of high quality of *Populus*.

The *GT31B-1* is the target of miR403b in *Populus* but is absent in *Arabidopsis*, suggesting that miR403b has different functions in these species. Hence, to improve the understanding of Pto-miR403b, we dissected the effects of allelic variation in *PtoGT31B-1*. Due to the putative function of *PtoGT31B-1* is implicated in the biosynthesis of cell wall components, we focused on the genetic effects in wood formation. We found 10 SNPs in *PtoGT31B-1* associated with nine wood characteristic traits, including the five associated with *PtoMIR403b*, implying that the function of PtomiR403b depends on the target and that PtomiR403b-GT31B-1 shares a wood-formation regulatory network. Interestingly, PtoGT31B-1_SNP133 was significantly associated with different genetic effects on four traits, indicating the pleiotropy of *PtoGT31B-1* in wood formation.

The epistatic effect is an important genetic component contributing to the genetic architecture of quantitative traits (Phillips, 2008; Mackay, 2014). Previous studies have shown that the most traits are a function of the actions of more than one gene or locus, particularly continuous variation traits, which are consequences of the actions of multiple loci (Roff and Emerson, 2006). Only three SNPs were identified in both single SNP-association and epistasis analyses, implying that epistatic interactions of multiple SNPs have complementary effects on a given trait. For example, PtoMIR403b_SNP38 interacted with 13 SNPs contributing to four wood characteristic traits and did not exhibit significantly additive or dominant effects. Regardless of the epistasis is the important effects underlying the desirable trait, it is still considered as a nuisance and ignored in plant breeding (Roff and Emerson, 2006; Sackman and Rokyta, 2018). Therefore, the incorporation of epistasis in breeding programs is challenging. VanRaden (2008) have first attempts to consider the epistasis in genomic selection, and several models have been developed to improve the prediction accuracy (Jiang and Reif, 2015). Additionally, the epistatic effect shapes phenotypic variation during domestication selection, suggesting an important role for gene-gene interactions in complex plant traits (Doust et al., 2014). We integrated additive, dominant, and epistatic effects to assess the genetic effects of *PtoMIR403b* and *PtoGT31B-1* on wood characteristic traits. The results provide insight into the role of PtomiR403b in wood formation and highlight several candidate SNPs for marker-assisted breeding of *Populus*.

### Genetic Interactions Between *PtoMIR403b* and *PtoGT31B-1* Underlying Wood Properties

It is by now well established that miRNAs are dependent on their target genes (Chen, 2005; Chekulaeva and Filipowicz, 2009). Currently, a large number of evidence supports that the idea that miRNAs are involved in a broad spectrum of biological progresses through negative post-transcriptional gene regulation (Cai et al., 2009). Thus, only as target gene is confirmed will it be potential to establish commonalities that will enable more precisely predict miRNA-gene (Kuhn et al., 2008). A single miRNA may regulate hundreds target genes, thus it is a challenging to prove its function and interaction of miRNA-targets based on reverse genetics; however, association studies may provide a more relevant evaluation of the relationship between miRNA-targets.

Our results provide a robust evidence that the genetic interaction between *PtoMIR403b* and *PtoGT31B-1* underlies wood characteristic traits. The single SNP associations have revealed that *PtoMIR403b* and *PtoGT31B-1* were both associated with the wood characteristic traits and might be share the common functions in wood formation, and *PtoGT31B-1* was identified as a target of PtomiR403b. Epistasis is an interaction effect among multiple variants or genes that contribute to the same traits, and its analysis can reveal genetic interactions between two SNPs or genes underlying complex traits. We previously used the epistasis association study to construct a genetic interaction network for photosynthetic traits and revealed the allelic combinations among non-coding RNAs and protein-coding gene, suggesting the power of epistatic effects to illustrate the genetic interaction between miRNA and its target genes (Xiao et al., 2020). We identified 36 SNP-SNP pairs representing the interactions between *PtoMIR403b* and *PtoGT31B-1* and clarified how these interactions contribute to wood characteristic traits (Figure 3), revealing that *PtoMIR403b* and *PtoGT31B-1* interact and are involved the same regulatory pathway.

In addition, we also introduced the MR test to prove the causal relationship between *PtoMIR403b*, *PtoGT31B-1* and wood property traits, which uncovered the *PtoMIR403b* modulated the wood formation through regulate the expression of *PtoGT31B-1*. Conventionally, the MR test that uses genetic variants associated with a modifiable exposure level or intermediates to assess the causal relationship between variables and final outcomes (phenotypes) (Smith and Ebrahim, 2004; Evans and Davey, 2015). For instance, Su et al. (2021) investigated the genetic effects of SNP loci on rice yield using its component traits, suggesting the MR will be helpful for understanding the genetic basis of complex traits. Recently, the gene expression is regarded as an intermediate molecular phenotype that links genetic variants to plant traits. For example, MR test have prioritized 97 genes associated with drought tolerance and revealed that local variants regulate *abh2* expression and are negatively associated with drought tolerance in maize, indicating that gene expression bridges the genetic variation to phenotype (Liu et al., 2020). Moreover, miRNA as a *trans*-regulator modulate the expression of its target genes. Herein, we found that SNPs in precursors altered the stem-loop structure of PtomiR403b and were associated with target expression. Therefore, MR testing can demonstrate how genetic variants in miRNAs affect phenotype via *trans-*regulating target gene expression. Therefore, combining association analysis and MR test is a feasible approach for dissection of the function and interaction of miRNA-targets from massive bioinformatics prediction results.

## Data Availability Statement

The datasets presented in this study can be found in online repositories. The names of the repository/repositories and accession number(s) can be found in the article/Supplementary Material.

## Author Contributions

DZ designed the conception and experiment, obtained funding and was responsible for this article. LX, LM, LY, JZ, BL, and MQ collected the data and conducted statistical analysis. LX wrote the manuscript. LM, WL, YF, and DW provided valuable suggestion for the manuscript. LM, LY, and QD revised the manuscript. All authors read and approved the manuscript.

## Conflict of Interest

The authors declare that the research was conducted in the absence of any commercial or financial relationships that could be construed as a potential conflict of interest.

## Publisher’s Note

All claims expressed in this article are solely those of the authors and do not necessarily represent those of their affiliated organizations, or those of the publisher, the editors and the reviewers. Any product that may be evaluated in this article, or claim that may be made by its manufacturer, is not guaranteed or endorsed by the publisher.

## Footnotes

1
http://plantgrn.noble.org/psRNATarget/

2
http://www.mirbase.org/

3
http://tree.bio.ed.ac.uk/software/figtree/

4
http://rna.tbi.univie.ac.at/cgi-bin/RNAWebSuite/RNAfold.cgi

## Supplementary Material

Supplementary Figure 1 Stem-loop structure of PtomiR403b with SNPs. Three SNPs in the precursor region of PtomiR403b affect the secondary structure and MFE.

Supplementary Figure 2 Phylogenetic tree of miR403 precursors. Left, unrooted maximum-likelihood tree. Bootstrap values are from 1,000 replicates. Right, alignment of mature miR403 sequences. Red, missing nucleotides. Precursor and mature sequences of miR403 were downloaded from miRbase.

Supplementary Figure 3 Decay of LD in *PtoMIR403b* and *PtoGT31B-1*. Pairwise correlations between SNPs (*r*^2^) plotted against the physical distance between SNP pairs. Red curves are non-linear regressions of *r*^2^ based on physical distance.

Supplementary Figure 4 Expression levels of PtomiR403b and *PtoGT31B-1* according to the genotype of the PtoMIR403b_SNP31 and PtoMIR403b_SNP33 haplotype.

Supplementary Table 1 Oligonucleotide sequences of the primers used in this study.

Supplementary Table 2 Common SNPs (minor allele frequency ≥ 5%) in *PtoMIR403b* and *PtoGT31B-1*.

Supplementary Table 3 Significant SNPs in candidate genes associated with growth and wood characteristics in the association population of *P. tomentosa*.

Supplementary Table 4 SNP pairs and their main effects among the two candidate genes in the association population of *P. tomentosa*.

Supplementary Table 5 Results of MR analysis for SNP, *PtoGT31B-1* expression, and wood characteristic traits.

Supplementary Method 1 Degradome sequencing.

Supplementary Method 2 SNP calling following resequencing of the association population.

## References

[B1] Addo-Quaye C. Miller W. Axtell M. J. (2009). Cleaveland: a pipeline for using degradome data to find cleaved small RNA targets. *Bioinformatics* 25 130–131. 10.1093/bioinformatics/btn604 19017659 PMC3202307

[B2] Basu D. Tian L. Debrosse T. Poirier E. Emch K. Herock H. (2016). Glycosylation of a fasciclin-like arabinogalactan-protein (sos5) mediates root growth and seed mucilage adherence via a cell wall receptor-like kinase (fei1/fei2) pathway in *Arabidopsis*. *PLoS One* 11:e145092. 10.1371/journal.pone.0145092 26731606 PMC4701510

[B3] Basu D. Wang W. Ma S. DeBrosse T. Poirier E. Emch K. (2015). Two hydroxyproline galactosyltransferases, galt5 and galt2, function in arabinogalactan-protein glycosylation, growth and development in *Arabidopsis*. *PLoS One* 10:e125624. 10.1371/journal.pone.0125624 25974423 PMC4431829

[B4] Bradbury P. J. Zhang Z. Kroon D. E. Casstevens T. M. Ramdoss Y. Buckler E. S. (2007). Tassel: software for association mapping of complex traits in diverse samples. *Bioinformatics* 23 2633–2635. 10.1093/bioinformatics/btm308 17586829

[B5] Budak H. Akpinar B. A. (2015). Plant mirnas: biogenesis, organization and origins. *Funct. Integr. Genomics* 15 523–531. 10.1007/s10142-015-0451-2 26113396

[B6] Burgess S. Foley C. N. Zuber V. (2018). Inferring causal relationships between risk factors and outcomes from genome-wide association study data. *Annu. Rev. Genomics Hum. Genet.* 19 303–327. 10.1146/annurev-genom-083117-021731 29709202 PMC6481551

[B7] Cai Y. Yu X. Hu S. Yu J. (2009). A brief review on the mechanisms of mirna regulation. *Genom Proteom Bioinf.* 7 147–154. 10.1016/S1672-0229(08)60044-3 PMC5054406 20172487

[B8] Cardon L. R. Bell J. I. (2001). Association study designs for complex diseases. *Nat. Rev. Genet.* 2 91–99. 10.1038/35052543 11253062

[B9] Chekulaeva M. Filipowicz W. (2009). Mechanisms of mirna-mediated post-transcriptional regulation in animal cells. *Curr. Opin. Cell Biol.* 21 452–460. 10.1016/j.ceb.2009.04.009 19450959

[B10] Chen X. (2005). Microrna biogenesis and function in plants. *FEBS Lett.* 579 5923–5931. 10.1016/j.febslet.2005.07.071 16144699 PMC5127707

[B11] Doust A. N. Lukens L. Olsen K. M. Mauro-Herrera M. Meyer A. Rogers K. (2014). Beyond the single gene: how epistasis and gene-by-environment effects influence crop domestication. *Proc. Natl. Acad. Sci. U S A.* 111 6178–6183. 10.1073/pnas.1308940110 24753598 PMC4035984

[B12] Du Q. Xu B. Gong C. Yang X. Pan W. Tian J. (2014). Variation in growth, leaf, and wood property traits of chinese white poplar (populus tomentosa), a major industrial tree species in northern china. *Can. J. Forest Res.* 44 326–339. 10.1139/cjfr-2013-0416 33356898

[B13] Du Q. Yang X. Xie J. Quan M. Xiao L. Lu W. (2019). Time-specific and pleiotropic quantitative trait loci coordinately modulate stem growth in Populus. *Plant Biotechnol. J.* 17 608–624. 10.1111/pbi.13002 30133117 PMC6381792

[B14] Ehrenreich I. M. Purugganan M. D. (2008). Sequence variation of micrornas and their binding sites in *Arabidopsis*. *Plant Physiol.* 146 1974–1982. 10.1104/pp.108.116582 18305205 PMC2287364

[B15] Evans D. M. Davey S. G. (2015). Mendelian randomization: new applications in the coming age of hypothesis-free causality. *Annu. Rev. Genomics Hum. Genet.* 16 327–350. 10.1146/annurev-genom-090314-050016 25939054

[B16] Fagundes Lopes F. J. Pauly M. Brommonshenkel S. H. Lau E. Y. Diola V. Passos J. L. (2010). The egmur3 xyloglucan galactosyltransferase from eucalyptus grandis complements the mur3 cell wall phenotype in *Arabidopsis thaliana*. *Tree Genet Genomes* 6 745–756. 10.1007/s11295-010-0288-8

[B17] Fan D. Li C. Fan C. Hu J. Li J. (2020). Microrna6443-mediated regulation of ferulate 5-hydroxylase gene alters lignin composition and enhances saccharification in Populus tomentosa. *New Phytol.* 226 410–425. 10.1111/nph.16379 31849071

[B18] German M. A. Pillay M. Jeong D. H. Hetawal A. Luo S. Janardhanan P. (2008). Global identification of microrna-target rna pairs by parallel analysis of RNA ends. *Nat. Biotechnol.* 26 941–946. 10.1038/nbt1417 18542052

[B19] Hahn L. W. Ritchie M. D. Moore J. H. (2003). Multifactor dimensionality reduction software for detecting gene-gene and gene-environment interactions. *Bioinformatics* 19 376–382. 10.1093/bioinformatics/btf869 12584123

[B20] Hennet T. (2002). The galactosyltransferase family. *Cell Mol. Life. Sci.* 59 1081–1095. 10.1007/s00018-002-8489-4 12222957 PMC11337546

[B21] Jansson S. Douglas C. J. (2007). Populus: a model system for plant biology. *Annu. Rev. Plant Biol.* 58 435–458. 10.1146/annurev.arplant.58.032806.103956 17280524

[B22] Jensen J. K. Schultink A. Keegstra K. Wilkerson C. G. Pauly M. (2012). Rna-seq analysis of developing nasturtium seeds (*Tropaeolum majus*): identification and characterization of an additional galactosyltransferase involved in xyloglucan biosynthesis. *Mol. Plant* 5 984–992. 10.1093/mp/sss032 22474179 PMC3440008

[B23] Jiang Y. Reif J. C. (2015). Modeling epistasis in genomic selection. *Genetics* 201 759–768. 10.1534/genetics.115.177907 26219298 PMC4596682

[B24] Kong Y. Pena M. J. Renna L. Avci U. Pattathil S. Tuomivaara S. T. (2015). Galactose-depleted xyloglucan is dysfunctional and leads to dwarfism in *Arabidopsis*. *Plant Physiol.* 167 1294–1296. 10.1104/pp.114.255943 25673778 PMC4378170

[B25] Kuhn D. E. Martin M. M. Feldman D. S. Terry A. J. Nuovo G. J. Elton T. S. (2008). Experimental validation of mirna targets. *Methods* 44 47–54. 10.1016/j.ymeth.2007.09.005 18158132 PMC2237914

[B26] Kumar S. Stecher G. Tamura K. (2016). Mega7: molecular evolutionary genetics analysis version 7.0 for bigger datasets. *Mol. Biol. Evol.* 33 1870–1874. 10.1093/molbev/msw054 27004904 PMC8210823

[B27] Li C. Wang X. Ran L. Tian Q. Fan D. Luo K. (2015). PtoMYB92 is a transcriptional activator of the lignin biosynthetic pathway during secondary cell wall formation in *Populus tomentosa*. *Plant Cell Physiol.* 56 2436–2446. 10.1093/pcp/pcv157 26508520

[B28] Li Y. I. van de Geijn B. Raj A. Knowles D. A. Petti A. A. Golan D. (2016). RNA splicing is a primary link between genetic variation and disease. *Science* 352 600–604. 10.1126/science.aad9417 27126046 PMC5182069

[B29] Liu S. Li C. Wang H. Wang S. Yang S. Liu X. (2020). Mapping regulatory variants controlling gene expression in drought response and tolerance in maize. *Genome Biol.* 21:163. 10.1186/s13059-020-02069-1 32631406 PMC7336464

[B30] Lu S. Li Q. Wei H. Chang M. J. Tunlaya-Anukit S. Kim H. (2013). Ptr-miR397a is a negative regulator of laccase genes affecting lignin content in *Populus trichocarpa*. *Proc. Natl. Acad. Sci. U S A.* 110 10848–10853. 10.1073/pnas.1308936110 23754401 PMC3696765

[B31] Mackay T. F. (2014). Epistasis and quantitative traits: using model organisms to study gene-gene interactions. *Nat. Rev. Genet.* 15 22–33. 10.1038/nrg3627 24296533 PMC3918431

[B32] Matsumoto N. Takenaka Y. Wachananawat B. Kajiura H. Imai T. Ishimizu T. (2019). Rhamnogalacturonan i galactosyltransferase: detection of enzyme activity and its hyperactivation. *Plant Physiol. Biochem.* 142 173–178. 10.1016/j.plaphy.2019.07.008 31299599

[B33] Mishra P. J. Mishra P. J. Banerjee D. Bertino J. R. (2008). MiRsnps or miR-polymorphisms, new players in microrna mediated regulation of the cell: introducing microRNA pharmacogenomics. *Cell Cycle* 7 853–858. 10.4161/cc.7.7.5666 18414050

[B34] Neale D. B. Savolainen O. (2004). Association genetics of complex traits in conifers. *Trends Plant Sci.* 9 325–330. 10.1016/j.tplants.2004.05.006 15231277

[B35] Phillips P. C. (2008). Epistasis–the essential role of gene interactions in the structure and evolution of genetic systems. *Nat. Rev. Genet.* 9 855–867. 10.1038/nrg2452 18852697 PMC2689140

[B36] Porcu E. Rueger S. Lepik K. Santoni F. A. Reymond A. Kutalik Z. (2019). Mendelian randomization integrating gwas and eQTL data reveals genetic determinants of complex and clinical traits. *Nat. Commun.* 10:3300. 10.1038/s41467-019-10936-0 31341166 PMC6656778

[B37] Porth I. Klapste J. Skyba O. Hannemann J. McKown A. D. Guy R. D. (2013). Genome-wide association mapping for wood characteristics in Populus identifies an array of candidate single nucleotide polymorphisms. *New Phytol.* 200 710–726. 10.1111/nph.12422 23889164

[B38] Roff D. A. Emerson K. (2006). Epistasis and dominance: evidence for differential effects in life-history versus morphological traits. *Evolution* 60 1981–1990. 10.1111/j.0014-3820.2006.tb01836.x 17133855

[B39] Sackman A. M. Rokyta D. R. (2018). Additive phenotypes underlie epistasis of fitness effects. *Genetics* 208 339–348. 10.1534/genetics.117.300451 29113978 PMC5753867

[B40] Showalter A. M. Basu D. (2016). Extensin and arabinogalactan-protein biosynthesis: glycosyltransferases, research challenges, and biosensors. *Front. Plant Sci.* 7:814. 10.3389/fpls.2016.00814 27379116 PMC4908140

[B41] Smith G. D. Ebrahim S. (2004). Mendelian randomization: prospects, potentials, and limitations. *Int. J. Epidemiol.* 33 30–42. 10.1093/ije/dyh132 15075143

[B42] Su J. Xu K. Li Z. Hu Y. Hu Z. (2021). Genome-wide association study and mendelian randomization analysis provide insights for improving rice yield potential. *Sci. Rep.* 11:6894. 10.1038/s41598-021-86389-7 33767346 PMC7994632

[B43] van der Graaf A. Claringbould A. Rimbert A. Westra H. J. Li Y. Wijmenga C. (2020). Mendelian randomization while jointly modeling cis genetics identifies causal relationships between gene expression and lipids. *Nat. Commun.* 11:4930. 10.1038/s41467-020-18716-x 33004804 PMC7530717

[B44] VanRaden P. M. (2008). Efficient methods to compute genomic predictions. *J. Dairy Sci.* 91 4414–4423. 10.3168/jds.2007-0980 18946147

[B45] Wegrzyn J. L. Eckert A. J. Choi M. Lee J. M. Stanton B. J. (2010). Association genetics of traits controlling lignin and cellulose biosynthesis in black cottonwood (*Populus trichocarpa*. Salicaceae) secondary xylem. *New Phytol.* 188 515–532. 10.1111/j.1469-8137.2010.03415.x 20831625

[B46] Xiao L. Quan M. Du Q. Chen J. Xie J. Zhang D. (2017). Allelic interactions among *Pto-MIR475b* and its four target genes potentially affect growth and wood properties in *Populus*. *Front. Plant Sci*. 8:1055. 10.3389/fpls.2017.01055 28680433 PMC5478899

[B47] Xiao L. Liu X. Lu W. Chen P. Quan M. Si J. (2020). Genetic dissection of the gene coexpression network underlying photosynthesis in Populus. *Plant Biotechnol. J.* 18 1015–1026. 10.1111/pbi.13270 31584236 PMC7061883

[B48] Xie J. Yang X. Song Y. Du Q. Li Y. Chen J. (2017). Adaptive evolution and functional innovation of populus-specific recently evolved microRNAs. *New Phytol.* 213 206–219. 10.1111/nph.14046 27277139

[B49] Yavorska O. O. Burgess S. (2017). Mendelianrandomization: an r package for performing mendelian randomization analyses using summarized data. *Int. J. Epidemiol.* 46 1734–1739. 10.1093/ije/dyx034 28398548 PMC5510723

[B50] Yu L. Chen H. Sun J. Li L. (2014). PtrKOR1 is required for secondary cell wall cellulose biosynthesis in Populus. *Tree Physiol.* 34 1289–1300. 10.1093/treephys/tpu020 24728296

[B51] Zhang J. Nieminen K. Serra J. A. Helariutta Y. (2014). The formation of wood and its control. *Curr. Opin. Plant Biol.* 17 56–63. 10.1016/j.pbi.2013.11.003 24507495

[B52] Zhang H. Guo Z. Zhuang Y. Suo Y. Du J. Gao Z. (2021). MicroRNA775 regulates intrinsic leaf size and reduces cell wall pectin levels by targeting a Galactosyltransferase gene in *Arabidopsis*. *Plant Cell.* 33 581–602. 10.1093/plcell/koaa049 33955485 PMC8136896

[B53] Zhong R. Ye Z. H. (2014). Complexity of the transcriptional network controlling secondary wall biosynthesis. *Plant Sci.* 229 193–207. 10.1016/j.plantsci.2014.09.009 25443846

