# Supplementary figures and images for: Association Study and Mendelian Randomization Analysis Reveal Effects of the Genetic Interaction Between PtoMIR403b and PtoGT31B-1 on Wood Formation in Populus tomentosa
Source: Front Plant Sci. 2021 Aug 30;12:704941. doi: 10.3389/fpls.2021.704941 (PMC8435637; doi:10.3389/fpls.2021.704941)

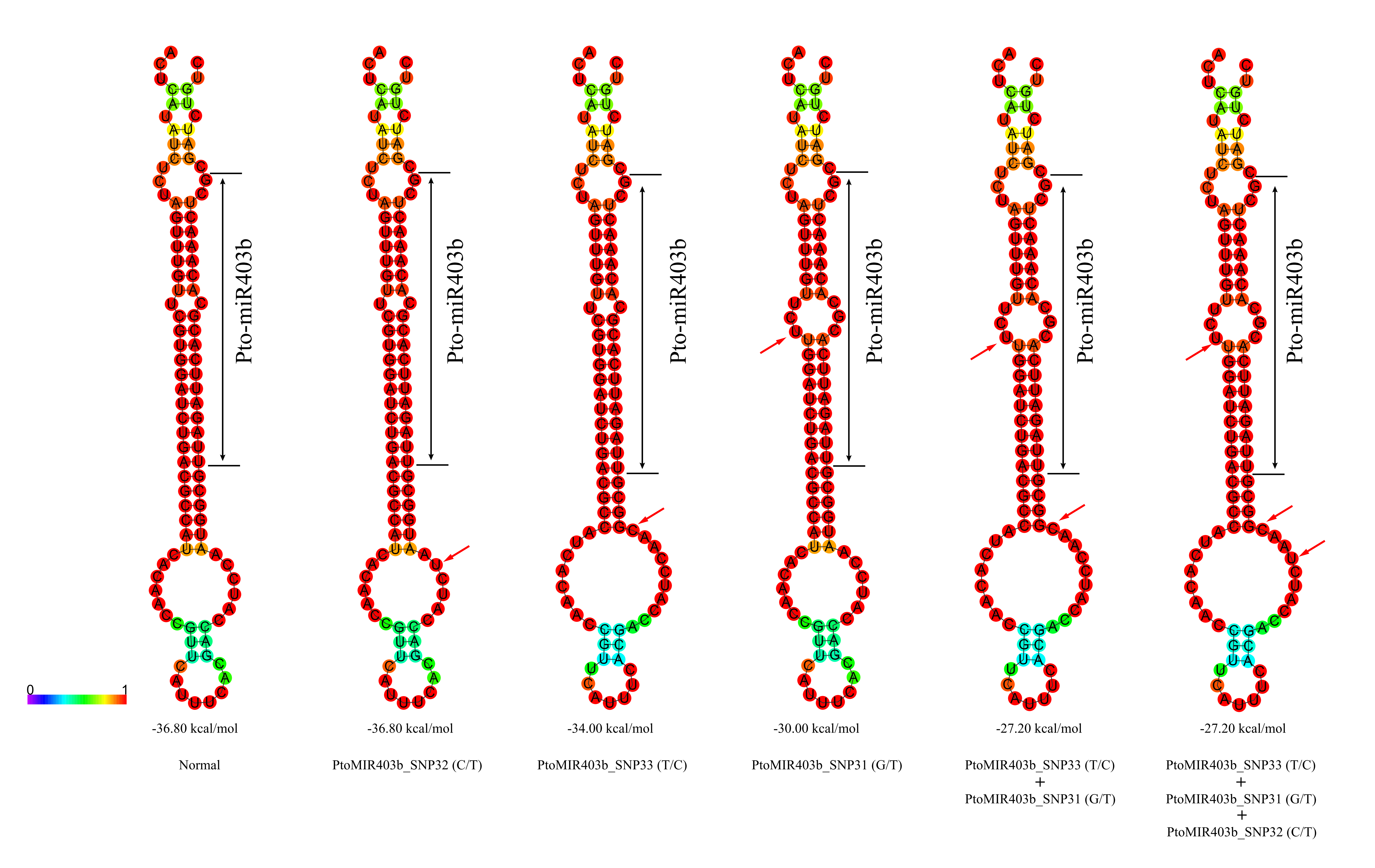

Supplement: Supplementary Figure 1 — Stem-loop structure of PtomiR403b with SNPs. Three SNPs in the precursor region of PtomiR403b affect the secondary structure and MFE. [file Image_1.TIF]

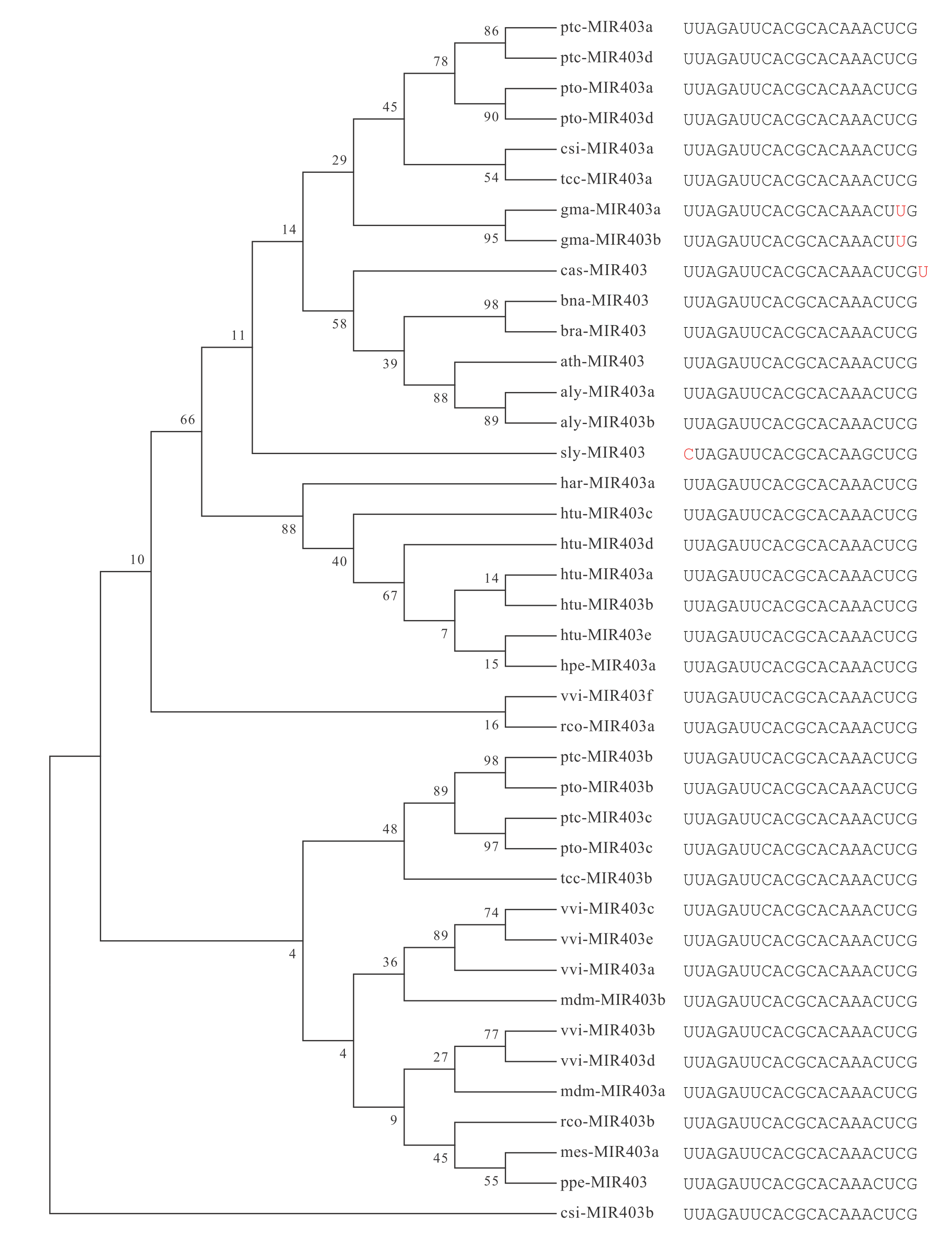

Supplement: Supplementary Figure 2 — Phylogenetic tree of miR403 precursors. Left, unrooted maximum-likelihood tree. Bootstrap values are from 1,000 replicates. Right, alignment of mature miR403 sequences. Red, missing nucleotides. Precursor and mature sequences of miR403 were downloaded from miRbase. [file Image_2.TIF]

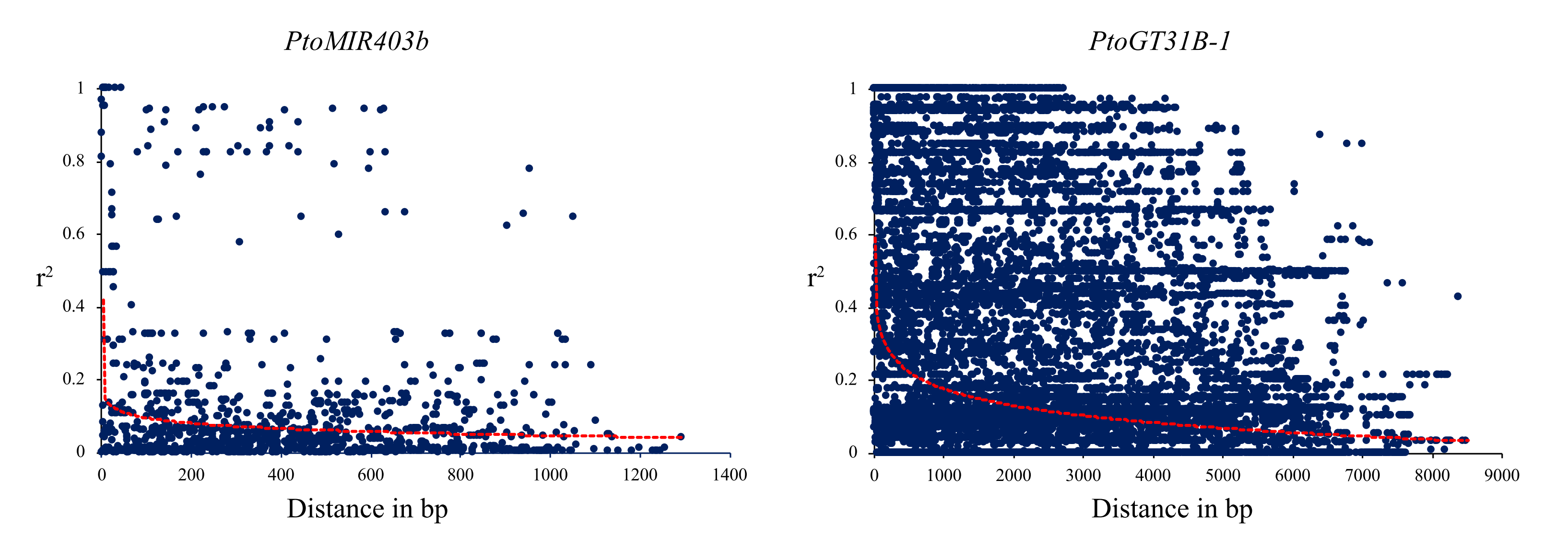

Supplement: Supplementary Figure 3 — Decay of LD in PtoMIR403b and PtoGT31B-1. Pairwise correlations between SNPs (r2) plotted against the physical distance between SNP pairs. Red curves are non-linear regressions of r2 based on physical distance. [file Image_3.TIF]

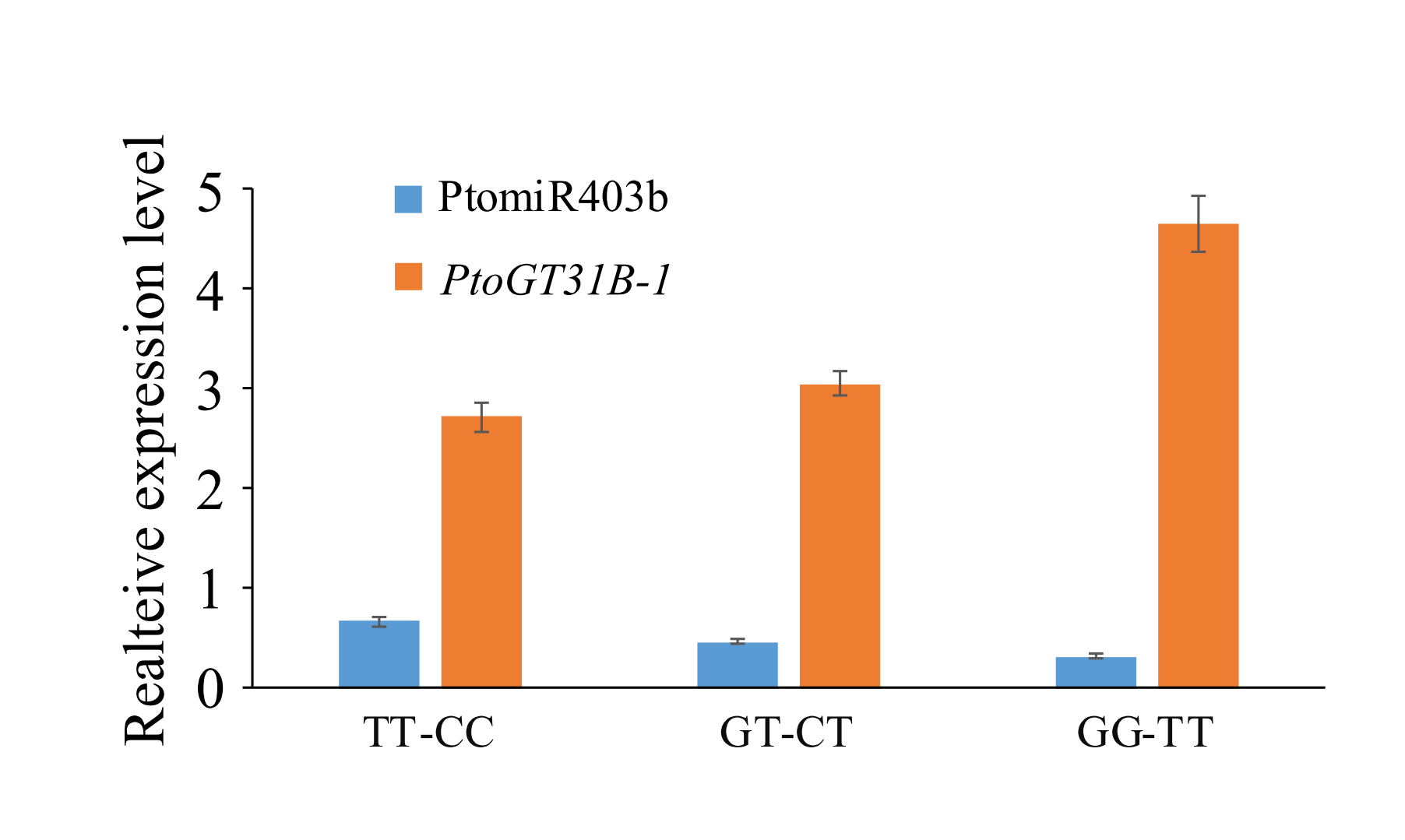

Supplement: Supplementary Figure 4 — Expression levels of PtomiR403b and PtoGT31B-1 according to the genotype of the PtoMIR403b_SNP31 and PtoMIR403b_SNP33 haplotype. [file Image_4.TIF]
