# Supplementary material for: Association Study and Mendelian Randomization Analysis Reveal Effects of the Genetic Interaction Between PtoMIR403b and PtoGT31B-1 on Wood Formation in Populus tomentosa
Source: Front Plant Sci. 2021 Aug 30;12:704941. doi: 10.3389/fpls.2021.704941 (PMC8435637; doi:10.3389/fpls.2021.704941)
Supplement: Supplementary Method 1 — Degradome sequencing. [file Data_Sheet_1.docx]

**Method S1 Degradome sequencing**

We performed degradome sequencing using equal pooled RNA samples of six tissues (leaf, shoot apex, phloem, cambium, developing xylem, and mature xylem) from *P. tomentosa*. The pooled RNA samples were used to construct degradome library as previously described ([Addo-Quaye et al., 2008](javascript:;)). In brief, poly(A^+^) RNA molecules were isolated from 200 µg of total RNA using the Oligotex mRNA kit (Qiagen), and then a 5’ RNA oligonucleotide adaptor containing an MmeI recognition site was ligated to the 5’-phosphate of the poly(A^+^) RNA by T4 RNA ligase. This was followed by purification of the ligated products using the Oligotex kit. Subsequently, five PCR cycles were performed on the products of a reverse transcription reaction which were then digested with MmeI and ligated to a 3’ double DNA adaptor. Finally, the ligation products were amplified with 20 PCR cycles, gel-purified, and subjected to SBS sequencing by the Illumina Genome analyzer.

The unique reads that perfectly matched *P. tomentosa* genome sequence were retained. Approximately 15 nt upstream and downstream of 5’ *P. tomentosa* transcript sequences, mapped by degradome reads, were extracted to generate 31 nt target signatures as ‘t-signatures’ ([German et al., 2008](javascript:;)), which were collected to align with the newly identified miRNAs and all mature known miRNAs from *Populus tomentosa* using the CleaveLand pipeline ([Addo-Quaye et al., 2009](javascript:;)). Alignments with scores up to four where G:U pairs scored 0.5 and no mismatches were found at the site between the 10th and 11th nucleotides of the corresponding miRNAs were considered potential targets.

**Method S2 SNP calling following resequencing of the association population**

For whole-genome resequencing, fresh leaves were collected from the 435 individuals of the *Populus tomentosa* association population, followed by total genomic DNA extraction using the DNeasy Plant Mini kit (Qiagen, Shanghai, China) following the manufacturer’s protocol. The extracted DNA was used for library construction according to the manufacturer’s recommendation (Illumina). Briefly, total DNA was randomly sheared into small fragments (200–300 bp) using a Covaris E210 ultrasonicator (Covaris, Inc., Woburn, MA, USA), followed by the overhangs converting into blunt ends using T4 DNA polymerase and DNA polymerase I Klenow fragment. After adenylation of 3′ ends of DNA fragments, Illumina adaptors (Illumina, San Diego, CA, USA) were then ligated to the ends of these DNA fragments using DNA ligase. DNA fragments that have adaptor molecules on both ends were selectively enriched using Illumina PCR Primer Cocktail and PCR products were purified (AMPure XP system) and quantified using the Agilent high-sensitivity DNA assay on the Agilent Bioanalyzer 2100 system. Finally, short-read sequence data were generated for each individual tree using an Illumina Genome Analyzer.

Raw reads were trimmed through a series of quality control (QC) procedures. QC standards were as follows: 1) removing reads with ≥10% unidentified nucleotides (N); 2) removing reads with >50% bases having phred quality <5; 3) removing reads with >10 nt aligned to the adapter, allowing ≤10% mismatches; and 4) removing putative PCR duplicates generated by PCR amplification in the library construction process (completely identical two paired-end reads).

Each sample clean reads were aligned to *P. tomentosa* reference genome by the Burrows-Wheeler Aligner (v0.7.5a-r405) using default parameters (Li and Durbin 2009). The low mapping quality (MQ <20) reads were filtered in SAMtools program (v1.1)(Li et al. 2009). Then, we performed genome variants calling by Genome Analysis Toolkit 4.0 (GATK 4.0, https://gatk.broadinstitute.org/hc/en-us) with conservative parameters (SNP: QD <5.0 || MQ <40.0 || FS >60.0 || SOR >3.0 || MQRankSum <-12.5 || ReadPosRankSum <-8.0). Finally, we pruned SNPs with only two alleles by Vcftools_0.1.13 (Danecek et al. 2011). Following, SNPs with not two alleles, a minor allele frequency (MAF) < 0.05 and a missing genotype > 0.2 were removed by VCFtools (Danecek et al. 2011).

**References:**

Addo-Quaye C. Eshoo TW. Bartel DP. Axtell MJ. 2008. Endogenous siRNA and miRNA targets identified by sequencing of the *Arabidopsis* degradome. Current Biology.18, 758–762.

Addo-Quaye C. Miller W. Axtell MJ. 2009. CleaveLand: a pipeline for using degradome data to find cleaved small RNA targets. Bioinformatics. 25, 130–131.

Danecek, P. A. Auton G. Abecasis, et al. 2011. The variant call format and VCFtools. Bioinformatics. 27:2156-8.

German MA. Pillay M. Jeong DH et al. 2008. Global identification of microRNA–target RNA pairs by parallel analysis of RNA ends. Nature Biotechnology26, 941–946.

Li H. and R. Durbin 2009. Fast and accurate short read alignment with Burrows-Wheeler transform. Bioinformatics. 25:1754-60.

Li H. B. Handsaker A. Wysoker et al. 2009. The Sequence Alignment/Map format and SAMtools. Bioinformatics. 25:2078-9.
